# Mortality Burden of the 2009 A/H1N1 Influenza Pandemic in France: Comparison to Seasonal Influenza and the A/H3N2 Pandemic

**DOI:** 10.1371/journal.pone.0045051

**Published:** 2012-09-20

**Authors:** Magali Lemaitre, Fabrice Carrat, Grégoire Rey, Mark Miller, Lone Simonsen, Cécile Viboud

**Affiliations:** 1 Fogarty International Center, National Institutes of Health, Bethesda, Maryland, United States of America; 2 Inserm, UMR-S 707, Paris, France; 3 UPMC – Paris 6, UMR-S 707, Paris, France; 4 Public Health Unit, Saint-Antoine hospital, AP-HP, Paris, France; 5 Inserm, CépiDC, Le Vésinet, France; 6 Department of Global Health, School of Public Health and Health Services, George Washington University, Washington, D.C., United States of America; University of Hong Kong, Hong Kong

## Abstract

**Background:**

The mortality burden of the 2009 A/H1N1 pandemic remains unclear in many countries due to delays in reporting of death statistics. We estimate the age- and cause-specific excess mortality impact of the pandemic in France, relative to that of other countries and past epidemic and pandemic seasons.

**Methods:**

We applied Serfling and Poisson excess mortality approaches to model weekly age- and cause-specific mortality rates from June 1969 through May 2010 in France. Indicators of influenza activity, time trends, and seasonal terms were included in the models. We also reviewed the literature for country-specific estimates of 2009 pandemic excess mortality rates to characterize geographical differences in the burden of this pandemic.

**Results:**

The 2009 A/H1N1 pandemic was associated with 1.0 (95% Confidence Intervals (CI) 0.2–1.9) excess respiratory deaths per 100,000 population in France, compared to rates per 100,000 of 44 (95% CI 43–45) for the A/H3N2 pandemic and 2.9 (95% CI 2.3–3.7) for average inter-pandemic seasons. The 2009 A/H1N1 pandemic had a 10.6-fold higher impact than inter-pandemic seasons in people aged 5–24 years and 3.8-fold lower impact among people over 65 years.

**Conclusions:**

The 2009 pandemic in France had low mortality impact in most age groups, relative to past influenza seasons, except in school-age children and young adults. The historical A/H3N2 pandemic was associated with much larger mortality impact than the 2009 pandemic, across all age groups and outcomes. Our 2009 pandemic excess mortality estimates for France fall within the range of previous estimates for high-income regions. Based on the analysis of several mortality outcomes and comparison with laboratory-confirmed 2009/H1N1 deaths, we conclude that cardio-respiratory and all-cause mortality lack precision to accurately measure the impact of this pandemic in high-income settings and that use of more specific mortality outcomes is important to obtain reliable age-specific estimates.

## Introduction

A novel influenza A/H1N1 virus was first isolated in April 2009 in North America and spread rapidly worldwide, leading the World Health Organization (WHO) to declare the first influenza pandemic of the 21st century in June 2009 [1]. Almost three years later, the mortality impact of the 2009 A/H1N1 pandemic remains poorly quantified in many countries, and comparisons with the burden of previous epidemic and pandemic seasons are scarce [2]–[6]. Early reports have suggested important geographical variation in pandemic disease burden globally; however methodological differences hamper fair comparisons between countries and seasons. Published mortality estimates for the 2009 pandemic suggest a relatively low impact in Europe [2], [7]–[9], Asia [5], and the US [4], and a moderate impact in Mexico [6]. Overall, all countries have consistently reported an increase of pandemic-related deaths in younger age groups, in contrast to inter-pandemic seasons in which 90% of deaths occur in seniors [4], [6]–[8], [10]–[12].

Methodological issues in the estimation of influenza disease burden make comparisons between countries and seasons difficult. Laboratory confirmation of influenza-related deaths is not conducted routinely. Although influenza testing was greatly strengthened during the pandemic, laboratory-confirmed pandemic deaths remain a gross underestimation of the overall influenza disease burden [10]. Deaths associated with influenza often occur following bacterial super-infection or aggravation of chronic diseases, after primary viral infection has been cleared. Consequently, death is often attributed to the underlying chronic condition rather than influenza [13]. It is now well accepted that the best approach to estimate influenza-related mortality is to apply statistical time series seasonal regression models to different causes of death [14]–[16].

Although estimates of pandemic mortality burden exist for Europe, they are mostly limited to all-cause mortality, a non-specific outcome, or to deaths coded specifically as influenza, which underestimates disease burden [2], [7], [8]. Moreover, no study has compared the impact of the 2009 pandemic with that of past pandemics using a similar methodology. The objectives of our study were to estimate the age- and cause-specific excess mortality rates of the first wave of the A/H1N1 pandemic in France, and compare pandemic estimates with those for seasonal epidemics and the A/H3N2 pandemic. We also reviewed published estimates of 2009 pandemic excess mortality rates in other countries for comparison purposes.

## Methods

### Data Sources

#### Mortality and population data

The A/H3N2 pandemic virus had its major impact in the 1969–70 winter in France, similarly to other countries in Europe [17]. Hence to study the impact of the A/H3N2 pandemic, the 2009 A/H1N1 pandemic, and seasonal epidemics, we obtained weekly age- and cause-specific mortality counts from June 1969 through May 2010 from national death certificates collected by Inserm CépiDc [18]. We categorized mortality based on the underlying cause of death, using the International Classification of Disease codes for influenza, pneumonia, respiratory diseases, cardiovascular diseases, and all causes (Text S1, Table S1). We used conversion factors published by CépiDc to account for transition between classifications in France [19].

We stratified deaths by 5 different age groups (0–4, 5–24, 25–44, 45–64, ≥65 yrs) and integrated yearly age-specific population data to calculate standardized death rates [20], using the June 2009 French population structure as reference.

#### Viral surveillance data

**Figure 1:**
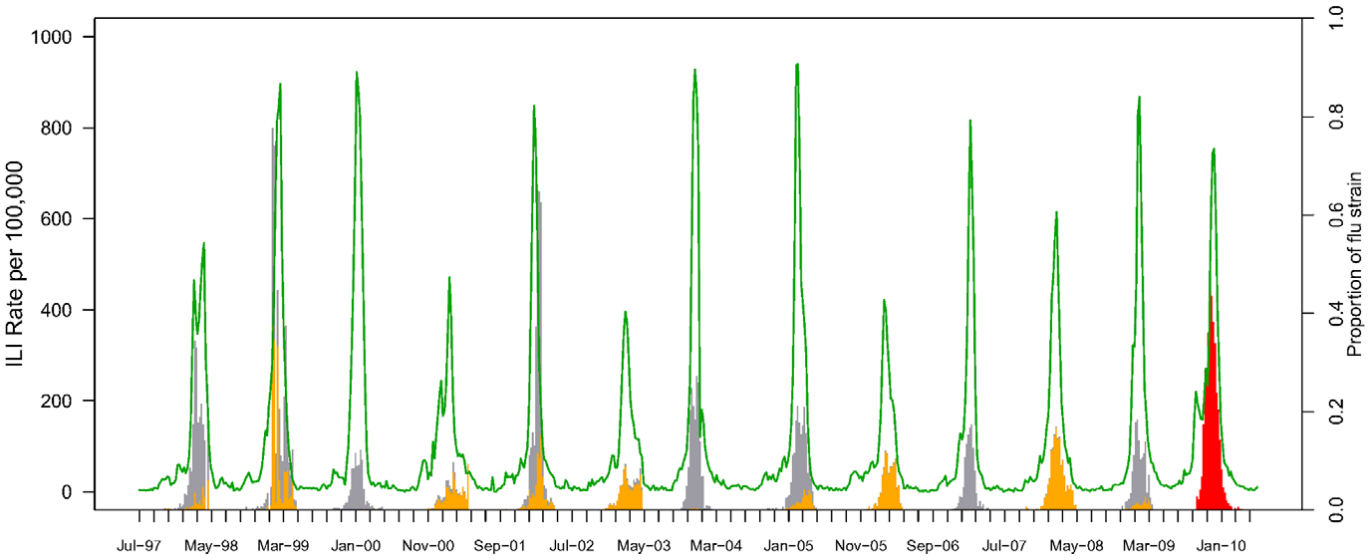
Weekly indicators of influenza activity, France, July 1997-June 2010. The green line represents the incidence of influenza like illnesses (ILI) per 100,000, as monitored by the French Sentinel Network. Vertical bars represent influenza virus activity, specifically the weekly percent positive influenza virus among all respiratory specimens tested, separately for each subtype (red for 2009 pandemic influenza A/H1N1, orange for seasonal A/H1N1-B, grey for seasonal A/H3N2).

Weekly percent of respiratory specimens testing positive for influenza were obtained from the FluNet database for France from January 1997 to June 2010, separately for each subtype (seasonal A/H1N1, B, A/H3N2 and pandemic A/H1N1; Figure 1) [21]. No viral surveillance data exist before 1997 in France. Influenza virus surveillance in France relies on a network of general practitioners [22], [23] and participating hospitals [24]. We considered an influenza subtype to be dominant when it accounted for at least 75% of all isolates subtyped in the respiratory season (July-June). When no single subtype accounted for more than 75% of all isolates, we summed the contribution of the two most prevalent subtypes.

#### Morbidity data

Influenza-like illness (ILI) has been shown to be a good proxy for influenza incidence in France and elsewhere [25], [26]. Weekly ILI incidence was obtained from the French Sentinel system, a nationwide network of general practitioners which has been reporting electronically the weekly number of medical visits for ILI and other infections since 1984 [27], [28]. One percent of all French practitioners voluntarily participate to disease surveillance through this system. The ILI case definition consisted of a combination of fever >39°C, myalgia, and respiratory symptoms. The French Sentinel Network ILI time series have been used in a number of previous epidemiological and modeling influenza studies aimed at detecting outbreaks, quantifying disease burden, and characterizing the spatio-temporal transmission dynamics of epidemics [26], [29]–[31].

### Excess Mortality Rates Estimation

#### Poisson model

The preferred approach to estimate influenza excess mortality is to explicitly link mortality from various causes to indicators of influenza activity, such as ILI or viral surveillance data [15], [32]. We used Poisson regression models with a log link [5] and considered both indicators of influenza activity [15], [32]. Models were fit to data for 1997–2010, a period when both ILI and viral surveillance data were available. A detailed description of the model fitting procedure is provided in Text S1 and summarized below.

First, we selected the indicator of influenza activity that provided the best statistical fit to the mortality data. We considered 3 potential indicators: weekly ILI incidence [26], weekly influenza virus percent positive [15], or their combination [25], [33]. [We tested various lags and moving averages of influenza indicators to account for delays between disease onset and death (Text S1). We selected the best influenza indicator and associated lag based on the Akaike Information Criterion (AIC) of respiratory death models. We focus on all-age respiratory death outcomes for this selection procedure because respiratory mortality has intermediate sensitivity and specificity to capture influenza mortality burden. We also checked that the selected influenza activity indicator maximized the correlation between observed and predicted values of respiratory deaths.

Second, we applied Poisson models using the influenza indicator selected by the above procedure to age- and cause-specific mortality time series. A stepwise selection method was used to identify the significant time trends and seasonal terms in the age- and cause-specific models (See Text S1 for full model equation).

Baseline mortality was calculated from the Poisson model as the expected values when influenza activity indicators are set to zero. Excess death rates due to influenza were estimated as the difference between predicted mortality from the full model and baseline mortality. The seasonal number of excess deaths due to influenza was summed for each respiratory season and age-standardized seasonal excess mortality rates were calculated. Confidence intervals were based on the variance of parameter estimates (See Text S1).

#### Serfling model

As a sensitivity analysis, we compared excess mortality estimates derived from the Poisson approach to those derived from a traditional Serfling model that does not include indicators of influenza activity, for the same time period, 1997–2010 (details of Serfling model are provided in Text S1 and [14], [34], [35]).

Given the unavailability of ILI and viral surveillance data in the years surrounding the historical A/H3N2 pandemic, we also used a Serfling approach to estimate the mortality impact of this pandemic.

## Results

### Influenza Virus Activity, 1997–2010

The A/H3N2 subtype was dominant in 7 of the 12 inter-pandemic seasons studied in France, 1997–2010, A/H1N1 and B subtypes were dominant or co-dominant in 4, and there was mixed A/H3N2-B circulation in one season. The A/H1N1 pandemic virus was first isolated on May 1st, 2009 based on the national influenza surveillance system [36]. Increased community transmission of pandemic A/H1N1 started in September 2009, virus activity peaked in the week of November 22, 2009, and A/H1N1 virus remained dominant until February 21, 2010 (Figure 1). A sharp rise in ILI activity coincided with the pandemic period and peaked in the last week of November 2009. Hereafter, we consider the 2009 A/H1N1 pandemic season in France to run from May 1, 2009 to May 1, 2010.

### Excess Mortality Model Selection

The best model for respiratory deaths included a 2-week lagged moving average (t-2, t-1 and t) of ILI incidence. Based on AIC, this model was better than models using the proportion of virus positive or ILI multiplied by the proportion of virus positive.

**Figure 2:**
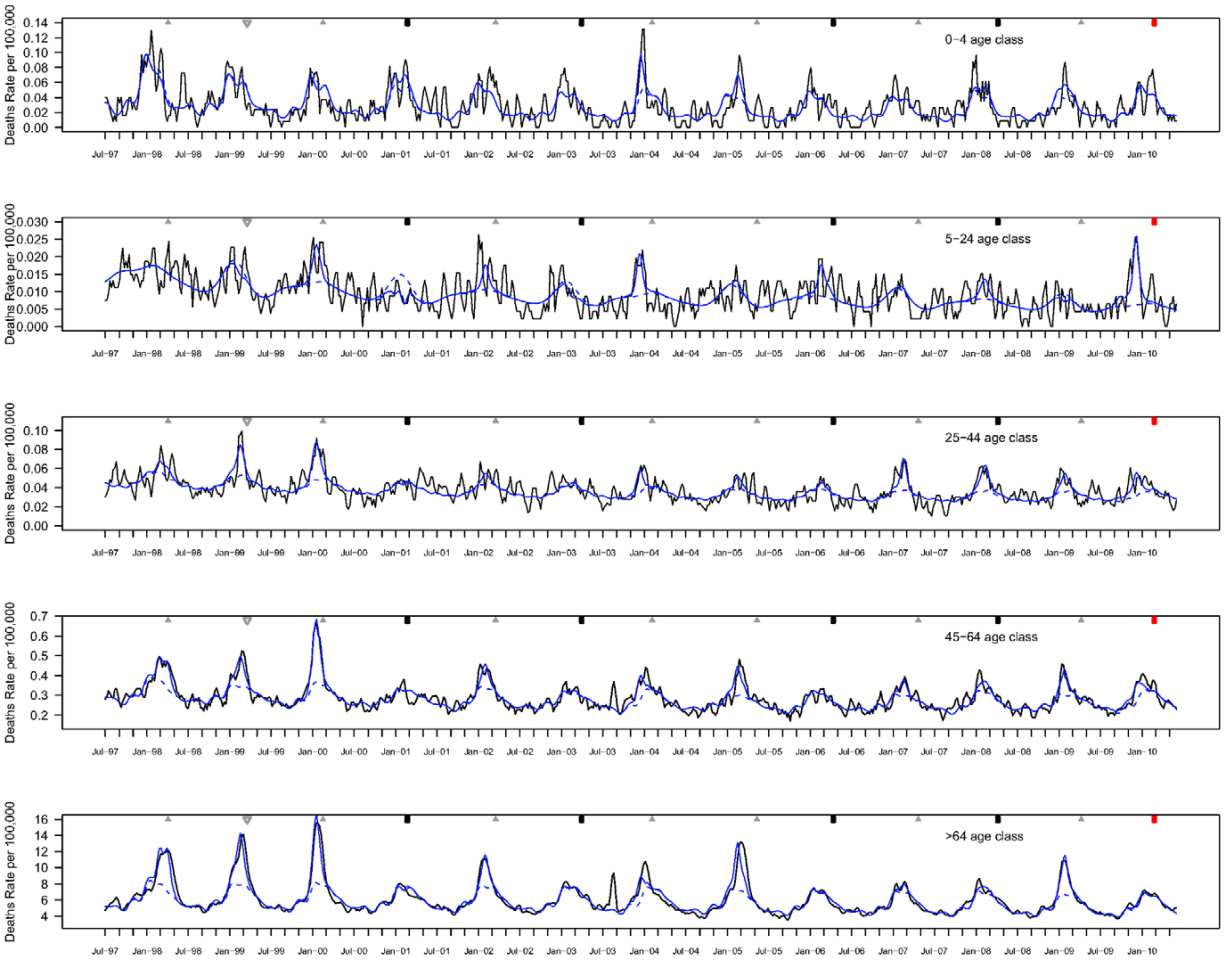
Weekly respiratory death rates by age group, France, July 1997-June 2010. Observed death rates (black line) and predicted death rates (blue line) by a Poisson model integrating seasonal terms, time trends and ILI data. Baseline mortality rates predicted by the Poisson model in the absence of influenza activity are indicated by a dashed blue line. Death rates were age-standardized using the 2009 French population as reference.

A good fit was achieved for most age groups and causes of death (Pearson correlation>0.56). Model fit was poorer for younger age groups (especially the 0–4 yrs; Figure 2, Text S1 and Figure S1). Model-predicted excess mortality peaks due to P&I, respiratory and cardio-respiratory were synchronous with ILI peaks (correlation between peak weeks: r>0.86, p<.0001; average lag between peaks 2.9 weeks).

**Figure 3:**
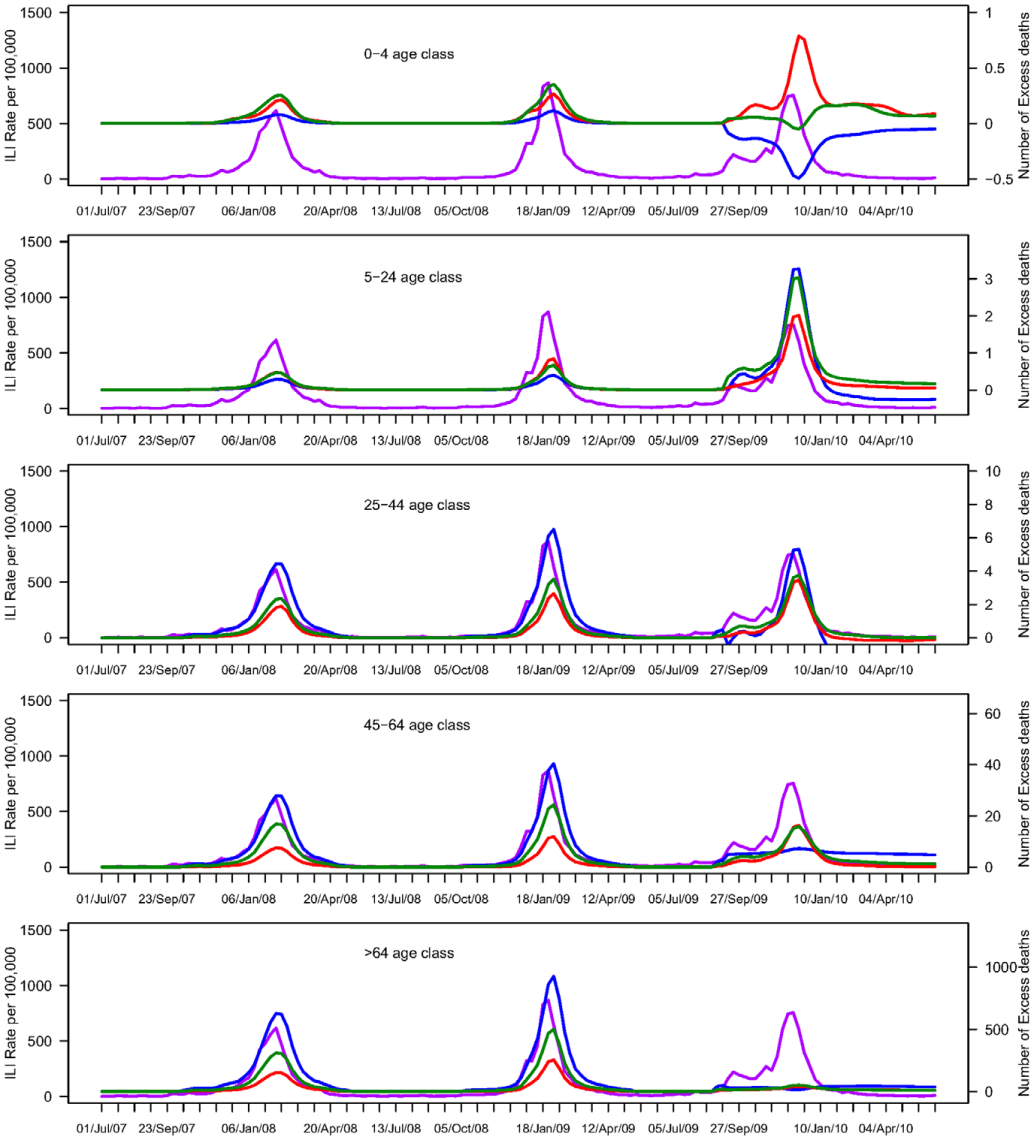
Weekly influenza like illnesses (ILI) overlaid with estimates of weekly numbers of influenza-related excess deaths by age group and outcome for the pandemic and previous seasons, France, 2008–2010. The purple line represents the incidence of influenza like illnesses (ILI) per 100,000. Red line represents excess mortality estimates for Pneumonia and influenza, green line is for respiratory mortality, and blue for cardio-respiratory outcomes. Excess deaths are predicted by a Poisson regression model integrating influenza activity data, time trends and seasonal terms.

As regards the A/H1N1 pandemic season, excess P&I and respiratory mortality rates peaked in November 2009 in all age groups, within 1 week of the peak in ILI and viral activity. Excess respiratory mortality increased substantially over baseline in age groups 5–64 years during the period of peak pandemic activity. This coincided with a small but significant increase in respiratory mortality in people over 65 years (Figure 3).

Peaks in excess cardio-respiratory mortality coinciding with pandemic activity were observed in age groups 5–44 yrs. In contrast, other age groups did not experience significant excess cardio-respiratory mortality (Figure 3, Table 1). Given the relatively low impact of the pandemic and the young age distribution of deaths, we concentrate below on respiratory outcomes, and provide estimates for other outcomes as relevant.

**Table 1:** Excess mortality rates associated with the 2009 A/H1N1 pandemic, the historical A/H3N2 pandemic, and seasonal epidemics by age group, death outcome, and disease burden model.

		Mortality outcomeRate per 100,000 (95% confidence interval)				
Season/Subtype	Age group		Pneumonia & Influenza	Respiratory	Cardio-respiratory	All causes
Pandemic A/H1N1 2009–10, based on Poisson model*	0–4		0.10 (0.04; 0.14)	0.02 (−0.17; 0.15)	−0.02 (−0.30; 0.21)	−0.71 (−2.0; 0.5)
	5–24		0.07 (0.06; 0.08)	0.13 (0.11; 0.15)	0.19 (0.14; 0.23)	0.36 (0.04; 0.67)
	25–44		0.15 (0.12; 0.18)	0.19 (0.13; 0.25)	0.29 (0.10; 0.48)	0.10 (−0.56; 0.75)
	45–64		0.80 (0.71; 0.89)	0.78 (0.55; 1.0)	0.43 (−0.31; 1.2)	−0.47 (−2.8; 1.8)
	>65		3.5 (1.2; 5.6)	4.1 (−0.94; 8.9)	−3.1 (−18; 11)	−6.59 (−41; 27)
	All ages		0.86 (0.43; 1.3)	0.98 (0.04; 1.9)	−0.28 (−3.0; 2.4)	−1.15 (−7.8; 5.4)
Pandemic A/H1N1 2009–10, based on Serfling model**	0–4		0.12 (0.02; 0.21)	0.17 (−0.02; 0.36)	0.28 (−0.06; 0.61)	0.74 (−1.1; 2.6)
	5–24		0.07 (0.04; 0.09)	0.10 (0.05; 0.15)	0.13 (0.03; 0.23)	0.39 (−0.12; 0.90)
	25–44		0.15 (0.10; 0.21)	0.21 (0.11; 0.31)	0.30 (0.01; 0.59)	0.74 (−0.20; 1.7)
	45–64		0.70 (0.54; 0.85)	0.75 (0.43; 1.1)	0.93 (0.05; 1.8)	2.7 (0.21; 5.2)
	>65		1.8 (−0.16; 3.7)	3.2 (−1.1; 7.8)	7.4 (−4.6; 19)	22 (−1.9; 45)
	All ages		0.54 (0.22; 0.87)	0.82 (0.09; 1.6)	1.6 (−0.41; 3.6)	4.7 (0.66; 8.7)
Seasonal A/H1N1-B based on Poisson model	0–4		0.04 (−0.06; 0.09)	0.07 (−0.11; 0.21)	0.00 (−0.31; 0.25)	0.48 (−0.76; 1.7)
	5–24		0.01 (−0.02; 0.02)	0.00 (−0.04; 0.03)	0.01 (−0.06; 0.07)	−0.12 (−0.47; 0.22)
	25–44		0.04 (−0.01; 0.08)	0.07 (−0.01; 0.13)	0.33 (0.13; 0.53)	−0.20 (−0.86; 0.45)
	45–64		−0.02 (−0.15; 0.10)	0.14 (−0.12; 0.39)	1.01 (0.25; 1.8)	2.3 (−0.02; 4.6)
	>65		−0.90 (−3.9; 1.9)	1.02 (−4;9; 6.6)	8.2 (−7.1; 23)	13 (−21; 46)
	All ages		−0.14 (−0.70; 0.37)	0.23 (−0.86; 1.3)	1.7 (−1.1; 4.5)	2.7 (−4.0; 9.2)
Seasonal A/H3N2 based on Poisson model*	0–4		0.03 (−0.09; 0.08)	0.05 (−0.12; 0.18)	−0.03 (−0.30; 0.19)	0.23 (−0.82; 1.2)
	5–24		0.04 (0.02; 0.05)	0.02 (−0.01; 0.05)	0.02 (−0.04; 0.07)	0.10 (−0.19; 0.37)
	25–44		0.10 (0.06; 0.12)	0.14 (0.08; 0.19)	0.30 (0.14; 0.46)	0.49 (−0.06; 1.0)
	45–64		0.42 (0.33; 0.50)	0.99 (0.79; 1.2)	2.0 (1.4; 2.6)	5.2 (3.3; 7.1)
	>65		15 (13.4; 17)	30 (25.9; 34)	65 (53; 76)	105 (78; 132)
	All ages		2.7 (2.4; 3.1)	5.4 (4.6; 6.1)	11 (9.2; 14)	19 (13.9; 24)
Pandemic A/H3N2 1969–70 based on Serfling model**	0–4		4.2 (3.9; 4.5)	5.5 (5.0; 6.1)	6.7 (6.0; 7.4)	9.0 (5.7; 12)
	5–24		0.73 (0.69; 0.77)	0.92 (0.84; 0.99)	1.1 (0.93; 1.2)	1.4 (0.40; 2.5)
	25–44		3.1 (3.1; 3.2)	4.4 (4.3; 4.6)	6.2 (5.7; 6.6)	9.6 (8.3; 11.0)
	45–64		21 (21; 22)	32.1 (31.5; 32.7)	50 (48; 52)	74 (70; 78)
	>65		152 (150; 155)	203 (197; 209)	316 (294; 339)	424 (386; 462)
	All ages		32 (32; 33)	44 (43; 45)	68 (64; 72)	94 (87; 100)

* Estimates are based on a Poisson regression model linking weekly mortality with weekly incidence of influenza like illness (average based on 4 A/H1N1–B epidemics and based on 7 A/H3N2 epidemics for seasonal epidemics).

** Estimates based on the traditional Serfling seasonal regression approach, which does not incorporate indicators of influenza activity.

### 2009–10 Pandemic Excess Mortality Estimates

#### All ages

We estimate that 613 excess respiratory deaths (95% Confidence Interval (CI): 125; 1,188) were attributable to the A/H1N1 pandemic period in France, May 2009-May 2010, corresponding to excess death rates of 0.98 (95% CI: 0.20; 1.9) per 100,000 population (table 1). The 2009–10 pandemic excess mortality rate is 2.8 fold lower than those of inter-pandemic influenza seasons. The proportion of respiratory deaths attributable to influenza was 5% for the 2009–10 A/H1N1 pandemic season, 2 times lower than for seasonal epidemics. A more specific outcome such as P&I excess mortality suggests a similarly low relative impact of the pandemic (Table 1). Less specific outcomes such as cardio-respiratory and all-cause mortality provided estimates even lower than those of respiratory outcomes, and most of these estimates were non-significant. Sensitivity analysis using a Serfling approach estimated that 514 (95% CI 55; 973)_excess respiratory deaths were attributable to the A/H1N1 pandemic, which is 16% lower than the estimate derived from the Poisson approach (table 1).

#### Age-specific estimates

The age group 5–24 yrs was the most severely affected by the 2009–10 A/H1N1 pandemic, relative to the impact of seasonal epidemics (figures 2 and 3). In this age group, A/H1N1 pandemic excess respiratory and cardio-respiratory death rates were 10.6 to 14- fold higher than those of seasonal epidemics and 2 to 4-fold higher than those of the most severe A/H3N2 epidemic in 1999–2000 (table 1, table S2). In age group 25–44 yrs, the A/H1N1 pandemic excess respiratory mortality rate was 1.9 -fold higher than for typical influenza seasons. In age groups 45–64 yrs, pandemic excess mortality rates were similar to or lower than those of typical A/H3N2 seasons, depending on the outcome.

Those over the age of 65 yrs were least affected by the A/H1N1 pandemic, relative to the impact of past seasons (Figure 3, Table 1), with an excess respiratory death rate 3.8 fold lower than for typical influenza seasons. The A/H1N1 pandemic estimate for cardio-respiratory outcome was non-significant in this age group. Respiratory estimates among children under 5 yrs were not significant for the pandemic season, but lacked significance in previous inter-pandemic seasons as well (table 1).

**Figure 4:**
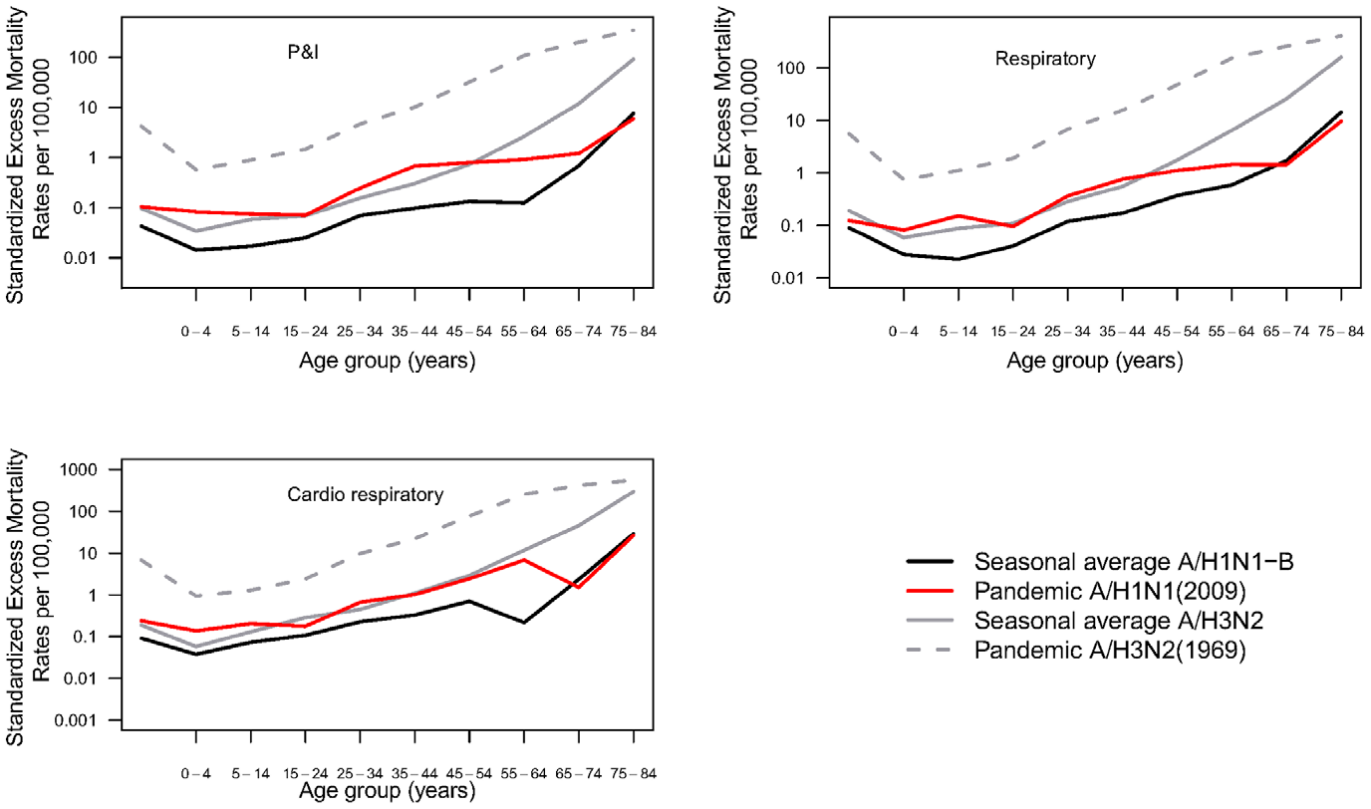
Age-specific excess death rates associated with the 2009–10 A/H1N1 pandemic, the 1969–70 A/H3N2 pandemic and seasonal A/H3N2 and A/H1N1-B epidemics, for different mortality outcomes. Excess mortality rates are estimated by a Serfling seasonal regression model, P&I: Pneumonia and influenza.

All cause excess mortality estimates were negative and non-significant in all age groups, except for the 5–24 yrs old. A more detailed age curve of influenza-related respiratory mortality based on 10-yr age groups suggests that individuals under 55 yrs experienced higher mortality rates in the A/H1N1 pandemic than in typical influenza seasons, while older individuals experienced mortality sparing (Figure 4).

In people aged 5–24 years old, the proportion of respiratory deaths attributable to A/H1N1 pandemic influenza was unusually high at 55%, even relative to the most severe H3N2 season in 1999–2000 (24%, P<0.03). Similarly, 28% of cardio-respiratory mortality were attributable to the pandemic in this age group, compared to only 7% in the most severe H3N2 season (p<0.03). Use of a more specific outcome, such as P&I, revealed similar age patterns, with 2.2-fold higher mortality impact during the pandemic among those aged 5–44 yrs, relative to seasonal epidemics, and almost 2-fold lower impact among seniors over 65 yrs (table 1).

We conducted several sensitivity analyses to test to robustness of our estimates to the choice of the link in Poisson models (identity, log), the definition of the pandemic period, and the choice of the modeling approach (Poisson vs Serfling). Estimates changes by less than 3% when using an identity link instead of a log link. Estimates were within 2% of those in the main analysis when using a stricter definition of the pandemic period limited to weeks when A/H1N1pdm was predominant, Sep 2009 to Feb 2010. Finally, sensitivity analyses using a Serfling approach confirmed the age patterns reported in the main analysis, with excess respiratory estimates within 0–23% of those derived from the Poisson model (Table 1 and S2).

### Comparison with the Historical A/H3N2 Pandemic

Based on the Serfling approach, we estimate that 27,600 and 42,600 excess respiratory and cardio-respiratory deaths respectively were attributable to the major A/H3N2 pandemic season in France in 1969–70 (rates of 44 and 68 deaths per 100,000 population, table 1). The proportions of deaths attributable to the A/H3N2 pandemic were 65% and 27% respectively for respiratory and cardio-respiratory mortality outcomes.

To provide a fair comparison of respiratory and cardio-respiratory mortality burden between the A/H3N2 and the 2009–10 A/H1N1 pandemics, we considered estimates derived from the same Serfling approach. The A/H3N2 pandemic was associated with substantially higher excess mortality impact than the A/H1N1 pandemic across all age groups and outcomes (Figure 4 and Table 1). Excess mortality rates were between 42 and 60-fold higher for the A/H3N2 pandemic than for the A/H1N1 pandemic depending on the outcome.

A mortality age shift was noted in both the A/H3N2 and A/H1N1 pandemics. While the proportions of excess respiratory deaths occurring in people under 65 years were 23% and 35% during the A/H3N2 and A/H1N1 pandemics, respectively, this age group accounted for only 7% of excess respiratory deaths during seasonal epidemics (p<0.0001). Analysis of cardio-respiratory outcomes suggests a similar age shift in both pandemic seasons (22% and 23% of excess deaths in people under 65 yrs in the A/H3N2 and A/H1N1 pandemics, respectively, vs. 6% during seasonal epidemics).

## Discussion

This is the first study to provide a systematic comparison of the age-specific mortality burden of the 2009–10 A/H1N1 pandemic with the historical A/H3N2 pandemic, based on detailed comparison of mortality outcomes and modeling approaches. Our burden estimates are based on a rigorous model selection approach considering several indicators of influenza activity. We estimate that the first season of circulation of the novel A/H1N1 pandemic virus in France (May 2009-May 2010) was associated with 0.98 excess respiratory deaths (95% CI: 0.20; 1.9) per 100,000. Relative to seasonal influenza, individuals aged 5–24 yrs were the most severely affected by the pandemic and experienced excess respiratory death rates 10.6 fold higher than those of seasonal epidemics. In contrast, the pandemic mortality burden in seniors over 65 years old was 3.8 fold lower than that of typical inter-pandemic seasons. The 2009–10 A/H1N1 pandemic was substantially milder than the A/H3N2 pandemic in France, consistently across all age groups and mortality indicators.

The French authorities identified 264 laboratory-confirmed pandemic A/H1N1 influenza deaths between July 1^st^, 2009 and February 28, 2010 [37] and 349 deaths listing an influenza code on the death certificate [36]. Our relatively conservative estimate of 613 pandemic excess respiratory deaths suggests that nearly 1 in 2 influenza-related deaths was laboratory confirmed, in a period where testing was considerably strengthened. For comparison purpose, a Mexican study suggested that only 1 in 7 influenza-related deaths were captured in laboratory-confirmed surveillance [6]. In France, the proportion of deaths occurring among seniors over 65 yrs was 26% based on laboratory-confirmed deaths [37], 44% based on deaths specifically coded as influenza [36] and 68% based on excess mortality models. The age distribution difference could be explained by a lesser propensity to confirm and diagnose influenza-related deaths among seniors than among younger individuals, as was reported for Mexico [6]. Alternatively, we could have overestimated excess respiratory death rates in seniors. However, a rise in P&I and respiratory mortality in this age group coincided with a period of intense pandemic activity in November-December 2009, lending validity to our estimates. In addition, we obtained a similar age distribution of influenza-related pandemic deaths by the Serfling approach (65% of excess respiratory deaths among seniors).

Overall, the mortality burden of the 2009–10 A/H1N1 pandemic in France was particularly mild, relative to the impact of seasonal influenza, except in the 5–44 yrs age group. Older populations did not experience significant excess cardio-respiratory and all cause mortality coinciding with the period of intense pandemic A/H1N1 activity. Most remarkably, the only age group to experience significant all-cause excess mortality rates was the 5–24 yrs, while all-cause estimates were negative in all other age groups, consistent with mortality patterns in other European countries [8], [38]. We therefore consider that P&I and respiratory deaths are the most reliable outcomes to model the age-specific mortality burden of the A/H1N1 pandemic in Europe and note that previously published all-cause mortality estimates from high-income countries in North America, Europe, and Asia may lack precision [4]–[6], [8], [9].

Our data confirm that the main waves of the A/H3N2 and A/H1N1 pandemics in France were characterized by mortality age shifts, with a significant increase in the proportion of excess respiratory deaths among people under 65 years during pandemics, relative to inter-pandemic seasons. Our results and those of others reinforce one of the signature features of influenza pandemics – a younger than usual age distribution of influenza-related deaths [11], [39]–[41]. In pandemic situations, a more appropriate estimation of disease burden may be the years of life lost approach, which integrates the age distribution of deaths with excess mortality estimates [42]. We applied the years of life lost approach to French data, revealing a 2–2.6 fold higher impact for the 2009 pandemic than for seasonal epidemics in people under 45 yrs, corroborating findings from other settings (table 2, [4], [6]).

**Table 2:** Year of Life Lost associated with the 2009–10 A/H1N1 pandemic, the 1969–70 A/H3N2 pandemic, and seasonal epidemics, by mortality outcome.

	Standardized Rate per 100,000 (95% Confidence intervals)		
	P&I	Respiratory	Cardio-respiratory
All age			
A/H1N1 2009–10	17 (11; 23)	19 (8; 33)	10 (3; 44)
Seasonal A/H1N1-B**	3 (1; 7)	8 (2; 20)	36 (9; 72)
Seasonal A/H3N2*	38 (32; 43)	74 (62; 86)	157 (125; 190)
A/H3N2 1969–70	559 (549; 569)	774 (752; 796)	1180 (1106; 1255)
Among persons under 45 years			
A/H1N1 2009–10	6 (4; 7)	8 (6; 11)	12 (6; 19)
Seasonal A/H1N1-B**	2 (1; 3)	3 (1; 6)	9 (3; 16)
Seasonal A/H3N2*	4 (2; 5)	4 (2; 7)	8 (3; 14)
A/H3N2 1969–70	125 (120; 130)	171 (161; 180)	222 (203; 242)

Estimates are given as rates per 100,000 population.

* based on 4 A/H1N1–B seasonal epidemics.

** based on 7 A/H3N2 seasonal epidemics.

P&I: Pneumonia and Influenza.

**Table 3:** Multinational comparison of excess mortality rates and age distribution of deaths associated with the 2009 A/H1N1 pandemic and seasonal epidemics, based on a literature review.

Country and data source	Excess mortality approach	Mortality outcome	2009 pandemic excess mortality ratesper 100,000 (95% CI)			Ratio of 2009 pandemic to seasonal epidemicmortality impact†			Proportion of 2009 pandemic excess deaths under 65 yrs
			All ages	Under 65 yrs	Over 65 yrs	All ages	Under 65 yrs	Over 65 yrs	
France (this study)	Poisson model *	Respiratory	0.98 (0.20; 1.9)	0.34 (0.23; 0.45)	4.1 (−0.94; 8.9)	0.34	1.6	0.26	0.30
United States [4] and [10]	Serfling model	All-cause	2.7; 15.6						
	Probability modeling approach	All-cause	4.1 (2.9; 6.0)	4.2 (3.0; 6.1)	4.4 (3.1; 6.5)	1.3	10.5	0.19	0.87
Mexico [6]	Serfling model	Respiratory	4.9 (4.0; 5.8)	1.1–6.6	11 (0.9; 20)	1.3	2.2	0.34	–
Hong Kong [5]	Poisson model **	Cardio-Respiratory	1.6 (0.4; 2.9)	–	16 (−10;41)	0.31	-	0.16	–
United Kingdom [8]	Poisson model **	All-cause	7.4	0.78	42	–	–	–	0.09
Denmark [38]	Poisson model **	All-cause	9.8 (7.4; 12.1)	<1.5 (0.02; 3.0)	53 (40; 66)	0.24	0.71	0.24	0.11
Netherlands [9]	Poisson model*	All-cause	3.7 (1.6; 5.8)	1.3 (0.53; 2.3)	16.6 (9.0; 32)	0.31	1.1	0.24	0.30
Australia [3]	Serfling model	All-cause	−6.0 (−12; −0.6)	<0.5 (−5; 5.9)	<−7.4 (−13; −2.0)	–	–	–	–

For studies providing analyses of cause-specific mortality, we report estimates based on respiratory outcomes, as we believe is the most reliable cause of death to estimate the burden of this pandemic. If respiratory estimates were not available, we report estimates from cardio-respiratory mortality, and when that was not available, from all-cause mortality.

CI: confidence intervals.

* Poisson model driven by ILI.

** Poisson model driven by viral activity.

† Calculated as [2009 pandemic excess mortality rate]/[average seasonal excess mortality rate].

There are several limitations to our study. First, we developed a Poisson regression model linking mortality with influenza activity data, which is a more specific approach to estimate influenza-related mortality than the traditional Serfling method [16]. However, this approach may be less widely applicable than the Serfling method in that it requires several years of reliable weekly viral activity data collected with systematic surveillance criteria, which is not always the case in pandemic situations [16]. Also, we obtained negative 2009–10 pandemic estimates for less specific outcomes in individuals over 45 yrs by the Poisson approach, while the Serfling method gave positive estimates, in particular for cardio-respiratory deaths. Another caveat relates to the use of ILI as a proxy of influenza activity in Poisson models. Sensitivity analyses identified ILI as the best covariate to model respiratory deaths, over any indicator relying on influenza virus surveillance data. The poorer statistical fit of models incorporating the influenza virus surveillance data, even after standardization, smoothing, and allowing for lags, could be related to age biases in influenza testing or lack of information on circulation of other respiratory viruses. Specifically, if the majority of virus specimens were collected from children, weekly viral surveillance activity could be asynchronous with weekly senior mortality rates and decrease model fit. Our models driven by ILI, which were selected based on statistical grounds, may not be the most biologically relevant in that they may include the contribution of various pathogens and overestimate the contribution of influenza. However, ILI data do not introduce an age bias if all age groups are represented and the probability of influenza infection given ILI is similar across age groups [43]. Most importantly, models driven by viral activity data suggest a similar pandemic mortality burden as models driven by ILI, with an excess mortality rate estimated at 0.93 per 100,000.

A third limitation relates to the fact that we did not include weekly indicators of respiratory syncytial virus (RSV) activity and other pathogens proxies in our models. Lack of RSV information could explain why our mortality estimates for seasonal influenza lack significance in children under 4 years, an age group where RSV predominates [15]. RSV-coded deaths were concentrated in January-March 2010 in France, suggesting that RSV activity did not overlap with the period of intense pandemic A/H1N1 virus activity in late fall. It is possible however that RSV circulation was displaced by pandemic activity [44], potentially affecting our estimate of baseline mortality in younger age groups. Similarly, the contribution of other seasonal factors such Streptococcus Pneumoniae and cold spells would be more important in winter, several weeks and months after pandemic activity had peaked [45]. Overall, many countries lack age-specific information on various respiratory pathogen activity; such data will be useful to improve excess mortality models in the future [46].

A final caveat of our study is the lack of adjustment on time trends in health care and treatment, which matter for comparison of mortality rates associated with recent and historical pandemics. During the historical A/H3N2 pandemic, pediatric intensive care units did not exist in some countries and some of the children who survived in 2009 would have probably succumbed during the earlier pandemic. Treatment of secondary bacterial infections [47] and complications has greatly improved since the 1960 s, together with widespread use of antivirals in the community and hospital settings, and occasional use of extracorporeal membrane oxygenation. Although our analyses did not adjust for these factors, the order of magnitude difference between mortality estimates for the two pandemics suggests factors that go beyond healthcare and treatment. The substantial severity of the A/H3N2 pandemic in France, relative to the 2009 A/H1N1 pandemic, could be explained by differences in virus virulence or prior immunity. The burden of the 2009 pandemic was likely mitigated by substantial cross protective immunity with previously circulating A/H1N1 viruses [6]–[10], [48], [49] and/or cross-subtypic immunity from previous exposure to A/H3N2. By contrast, immunity from vaccination was minimal in the first wave of the 2009 pandemic in most countries due to delays in vaccine production and delivery. Pandemic vaccination was initiated on November 2^nd^ 2009 in France, just 3 weeks before peak viral activity, and only 7.9% of the population was vaccinated by February 2010 [50].

Important between-country variation in the mortality burden of historical pandemics has been reported [17], [51]. Our study reveals the particularly high excess mortality impact of the A/H3N2 pandemic in France, relative to more recent epidemic or pandemic seasons. Previous work has highlighted the severity of the A/H3N2 pandemic in Western Europe, as compared with the Americas [17], [52]. To gauge geographical variation in 2009 pandemic mortality burden, we reviewed published national studies providing age-specific mortality estimates for the 2009–10 A/H1N1 season (Table 3). Despite between-country variations in absolute estimates, most countries reported lower all-age excess mortality burden for the 2009 pandemic than for recent seasonal epidemics. School-age children and young adults experienced atypical mortality increase in most countries, including the UK, US and Mexico [6], [8], [10], [38], [53], consistent with our data. Similar results were found by pooling all-cause mortality data from eight European countries or regions [53]. In contrast to other European studies, the Netherlands reported unusually high pandemic burden in children under 4 yrs [9]. All countries reported lower than expected mortality burden among seniors, with a ratio of pandemic to epidemic excess mortality rates ranging from 0.16–0.34 across 6 countries on 3 continents. We note however that of the 7 studies providing excess mortality estimates based on vital statistics [6], [8], [10], [38], [53], 5 studies focus on all-cause mortality. This is unfortunate because our study suggests that all-cause mortality may not provide the most precise estimates of 2009 pandemic burden, especially in Europe where the pandemic was particularly mild. Moreover, not all studies provide 95% confidence limits on the estimates, and methodologies differ between studies. Finally, little information on the mortality burden of past and recent pandemics is available from low-income countries of Asia and Africa, which is a key area for future research.

Our study adds to the growing body of evidence on the burden of the 2009 A/H1N1 pandemic and suggests a relatively low excess mortality impact in France compared to seasonal influenza, except in school-age children and young adults. Our results suggest that it is important to analyze cause-specific mortality outcomes, such as respiratory deaths, to accurately capture the burden of influenza in mild seasons, such as the 2009 pandemic. All-cause mortality models provided negative estimates for the pandemic in France, which is clearly imprecise as at least 264 laboratory-confirmed deaths are directly attributable to pandemic influenza A/H1N1. Estimates from many more settings, including low and middle income countries, are needed before the full spectrum of geographical variation in pandemic mortality burden can be established and the socio-economic and population determinants of disease burden are fully elucidated.

## Supporting Information

Figure S1 Weekly pneumonia and influenza death rates by age group, France, July 1997-June 2010. Observed death rates (black line) and predicted death rates (blue line) by a Poisson model integrating seasonal terms, time trends and influenza activity data. Baseline mortality rates predicted by the Poisson model in the absence of influenza activity are indicated by a dashed blue line. Death rates were standardized to the population of 2009.
(TIF)

Table S1 Codes used to identify mortality due to pneumonia and influenza, respiratory causes, respiratory and cardiac, and all causes in France, 1968–2010, based on the 8, 9 and 10^th^ revisions of the International Classification of Diseases (ICD). Underlying causes of death were considered. No important coding change occurred at the transition between ICD-8 and ICD-9 in France. To account for the transition from ICD-9 to ICD-10, we used conversion factors published by CépiDc in France [18].
(DOCX)

Table S2 Excess mortality rates associated with the severe 1999–2000 seasonal A/H3N2 epidemic by age group and death outcome. Estimates are based on the Serfling model.
(DOCX)

Text S1 Details on the models mortality: parameter estimates, model fitting procedure, calculation of the influenza-related mortality using Poisson models and Serfling method, and details on the years of life lost estimation.
(DOCX)
