# Supplementary figures and images for: Mortality Burden of the 2009 A/H1N1 Influenza Pandemic in France: Comparison to Seasonal Influenza and the A/H3N2 Pandemic
Source: PLoS One. 2012 Sep 20;7(9):e45051. doi: 10.1371/journal.pone.0045051 (PMC3447811; doi:10.1371/journal.pone.0045051)

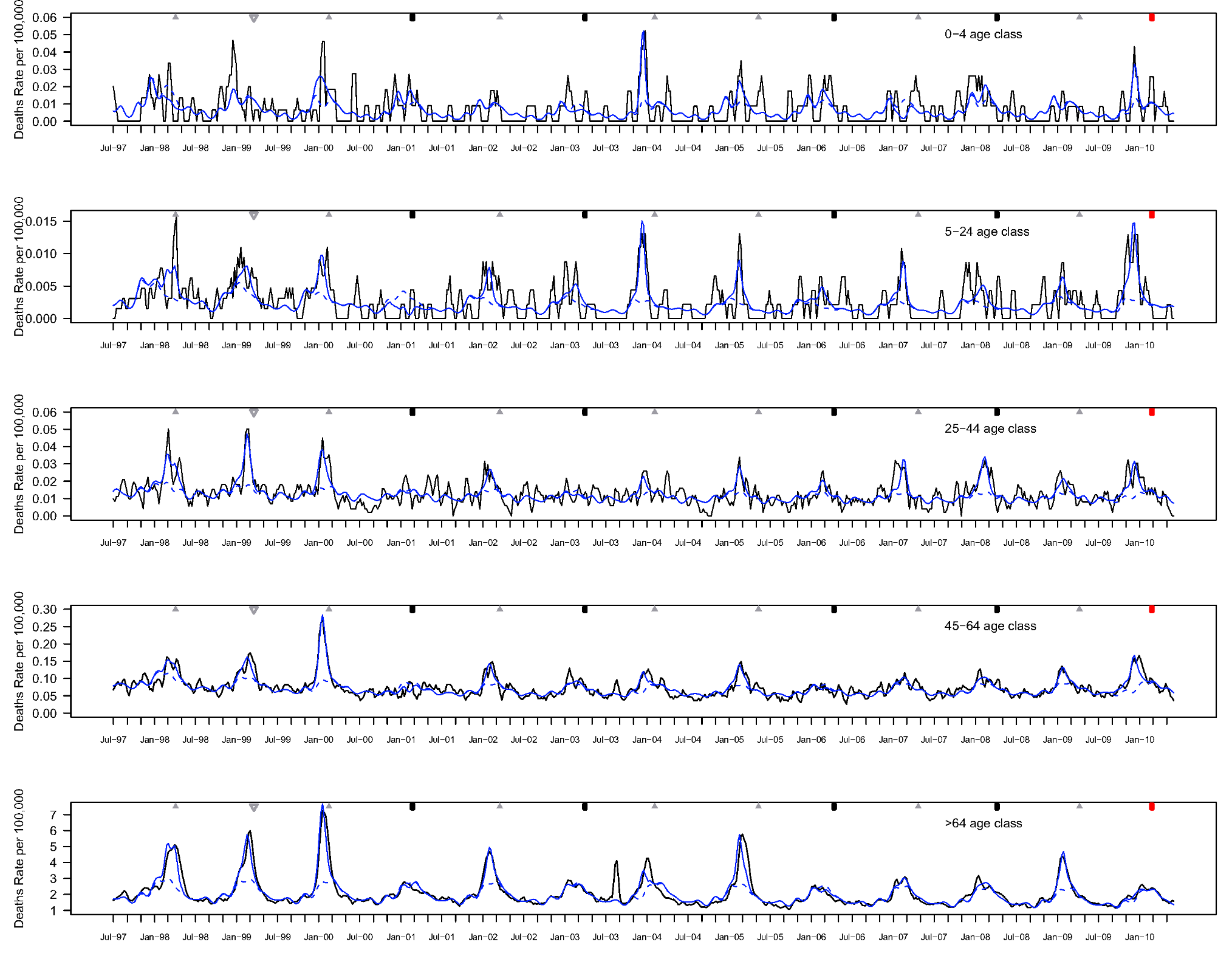

Supplement: Figure S1 — Weekly pneumonia and influenza death rates by age group, France, July 1997-June 2010. Observed death rates (black line) and predicted death rates (blue line) by a Poisson model integrating seasonal terms, time trends and influenza activity data. Baseline mortality rates predicted by the Poisson model in the absence of influenza activity are indicated by a dashed blue line. Death rates were standardized to the population of 2009. (TIF) [file pone.0045051.s001.tif]
