# Supplementary material for: Mortality Burden of the 2009 A/H1N1 Influenza Pandemic in France: Comparison to Seasonal Influenza and the A/H3N2 Pandemic
Source: PLoS One. 2012 Sep 20;7(9):e45051. doi: 10.1371/journal.pone.0045051 (PMC3447811; doi:10.1371/journal.pone.0045051)
Supplement: Table S1 — Codes used to identify mortality due to pneumonia and influenza, respiratory causes, respiratory and cardiac, and all causes in France, 1968–2010, based on the 8, 9 and 10th revisions of the International Classification of Diseases (ICD). Underlying causes of death were considered. No important coding change occurred at the transition between ICD-8 and ICD-9 in France. To account for the transition from ICD-9 to ICD-10, we used conversion factors published by CépiDc in France [18]. (DOCX) [file pone.0045051.s002.docx]

Table S1

Codes used to identify mortality due to pneumonia and influenza, respiratory causes, respiratory and cardiac, and all causes in France, 1968-2010, based on the 8, 9 and 10^th^ revisions of the International Classification of Diseases (ICD). Underlying causes of death were considered.

No important coding change occurred at the transition between ICD-8 and ICD-9 in France. To account for the transition from ICD-9 to ICD-10, we used conversion factors published by CépiDc in France [[18](#_ENREF_18)].

| **Cause of death** | **ICD** | **Year** | **Diseases Codes** |
| --- | --- | --- | --- |
| Influenza | 8 | 1968-78 | 470-474 |
|  | 9 | 1979-99 | 487 |
|  | 10 | 2000-2010 | J09-J11 |
| Pneumonia and Influenza | 8 | 1968-78 | 470-474, 480-486 |
|  | 9 | 1979-99 | 480-487 |
|  | 10 | 2000-2010 | J09-J18 |
| Respiratory | 8 | 1968-78 | 460-519 |
|  | 9 | 1979-99 | 460-519 |
|  | 10 | 2000-2010 | J00-J99 |
| Respiratory and Cardiac | 8 | 1968-78 | 390-519 |
|  | 9 | 1979-99 | 390-519 |
|  | 10 | 2000-2010 | I00-J99 |
| All causes | 8 | 1968-78 | All |
|  | 9 | 1979-99 | All |
|  | 10 | 2000-2010 | All |
