# Supplementary material for: Mortality Burden of the 2009 A/H1N1 Influenza Pandemic in France: Comparison to Seasonal Influenza and the A/H3N2 Pandemic
Source: PLoS One. 2012 Sep 20;7(9):e45051. doi: 10.1371/journal.pone.0045051 (PMC3447811; doi:10.1371/journal.pone.0045051)
Supplement: Table S2 — Excess mortality rates associated with the severe 1999–2000 seasonal A/H3N2 epidemic by age group and death outcome. Estimates are based on the Serfling model. (DOCX) [file pone.0045051.s003.docx]

Table S2

Excess mortality rates associated with the the severe 1999-2000 seasonal A/H3N2 epidemic by age group and death outcome. Estimates are based on the Serfling model.

|  |  | |  |  |  | |  |  | |  |  |  |
| --- | --- | --- | --- | --- | --- | --- | --- | --- | --- | --- | --- | --- |
|  |  | Mortality outcome  Rate per 100,000 (95% confidence interval) | | | | | | | | | | |
| Season/  Subtype | Age group | Pneumonia & Influenza | | | | Respiratory | | | Cardio-respiratory | | |  |
| Severe 1999-2000 Seasonal A/H3N2 | 0-4 | 0.16 (0.08 ; 0.23) | | | | 0.21 (0.05 ; 0.36) | | | 0.17 (-0.11 ; 0.44) | | |  |
|  | 5-24 | 0.04 (0.02 ; 0.06) | | | | 0.09 (0.05 ; 0.13) | | | 0.10 (0.01 ; 0.18) | | |  |
|  | 25-44 | 0.16 (0.11 ; 0.20) | | | | 0.29 (0.2 ; 0.37) | | | 0.48 (0.24 ; 0.71) | | |  |
|  | 45-64 | 1.0 (0.91 ; 1.2) | | | | 2.0 (1.8 ; 2.3) | | | 3.4 (2.7 ; 4.1) | | |  |
|  | >65 | 30 (28 ; 31) | | | | 55 (51 ; 59) | | | 106 (97 ; 116) | | |  |
|  | All ages | 5.3 (5.0 ; 5.6) | | | | 9.9 (9.3 ; 10.5) | | | 19 (17 ; 21) | | |  |
