# Supplementary material for: Mortality Burden of the 2009 A/H1N1 Influenza Pandemic in France: Comparison to Seasonal Influenza and the A/H3N2 Pandemic
Source: PLoS One. 2012 Sep 20;7(9):e45051. doi: 10.1371/journal.pone.0045051 (PMC3447811; doi:10.1371/journal.pone.0045051)
Supplement: Text S1 — Details on the models mortality: parameter estimates, model fitting procedure, calculation of the influenza-related mortality using Poisson models and Serfling method, and details on the years of life lost estimation. (DOCX) [file pone.0045051.s004.docx]

**Supporting text**

Magali Lemaitre*^1,2^, Fabrice Carrat^2,3,4^, Grégoire Rey^5^, Mark Miller^1^, Lone Simonsen^1,6^, Cécile Viboud^1^

^1^Fogarty International Center, National Institutes of Health, Bethesda, USA

^2^Inserm, UMR-S 707, Paris, France

^3^UPMC – Paris 6, UMR-S 707, Paris, France

^4^Public Health Unit, Saint-Antoine hospital, AP-HP, Paris, France

^5^Inserm, CépiDC, Le Vésinet, France

^6^Department of Global Health, George Washington University, Washington DC, USA

**Mortality models:**

***Poisson models***

In our main analysis, we fit Poisson models with a log link to weekly mortality data during 1997-2010. First, we selected the indicator of influenza activity that provided the best statistical fit to the mortality data. Three potential indicators of influenza activity were compared: weekly ILI incidence [[26](#_ENREF_25)], weekly influenza virus percent positive [[15](#_ENREF_15),21], and the combination of both [[25](#_ENREF_24)]. We also tried different lags and moving averages (0-3 weeks) to link mortality with influenza indicators. The Poisson model included terms for time trends, harmonics for seasonality, and weekly influenza indicators, as follows:

Full model:

Log(E(Y*_t/_Pop*)) = β_0_ + β_1_ X_flu(t)_ * Year_i + β_2*_t + β_3*_t^2^ + β_4_ t^3^ + β_5*_sin(2*Pi*t/52.17) + β_6*_cos(2*Pi*t/52.17) + β_7*_sin(3*Pi*t/52.17) + β_8*_cos(3*Pi*t/52.17) + β_9*_sin(4*Pi*t/52.17) + β_10*_cos(4*Pi*t/52.17) ) + β_11*_sin(6*Pi*t/52.17) + β_12*_cos(6*Pi*t/52.17) + β_13*_sin(8*Pi*t/52.17) + β_14*_cos(8*Pi*t/52.17) + β_15*_sin(10*Pi*t/52.17) + β_16*_cos(10*Pi*t/52.17) + β_17*_sin(12*Pi*t/52.17) + β_18*_cos(12*Pi*t/52.17)

Where Y_t_ represents weekly number of respiratory deaths for a specific age group and cause of death at week t, Pop is a population offset, t. t^2^ and t^3^ are linear, quadratic and cubic terms for time trend, sin and cos are harmonic terms representing seasonality, X_flu_ represents the lag or moving average of influenza activity indicator as explained below, and Year_i is a dummy variable representing each of the respiratory year studied, i=1…13. Specifically, in the models using viral activity data, X_flu(t)_ corresponds to the weekly proportion of respiratory specimens testing positive for each influenza subtype. In models using ILI morbidity data, X_flu_ corresponds to the weekly ILI incidence, ILI(t). In models combining influenza morbidity and viral surveillance, X_flu(t)_ corresponds to the incidence of ILI associated with each influenza subtype, ILI(t)*weekly_percent_positive_subtype(t)) as described in [[24](#_ENREF_24)]. Parameters (β_1-_ β_18_) were estimated using the maximum likelihood method.

We tried various lags and moving averages of influenza activity proxies to account for the delay between infection and death, Specifically, if flu(t) is a proxy for influenza activity at week t (ie, flu(t)= ILI(t), flu(t)=weekly_virus_percent_positive(t), or flu(t)=ILI(t)*weekly_percent_positive_subtype(t)), we tried:

a) 0-3 week lags, following :

X_flu(t)=flu(t), X_flu(t)=flu(t-1), X_flu(t)=flu(t-2), X_flu(t)=flu(t-3)

b) moving averages:

X_flu(t)=1/3(flu(t-1)+ flu(t)+flu(t+1))

X_flu(t)=1/3(flu(t-2)+ flu(t-1)+flu(t))

Selection of the most statistically meaningful proxy for influenza activity was based on fitting 18 models (3 proxies for influenza activity * 6 lag and moving average options) to all-age respiratory deaths and comparing Aikake values. We obtained the smallest AIC values for a moving average of ILI(t-2), ILI(t-1) and ILI(t) with an AIC of 4,175. For reference, the best model using weekly influenza virus percent positive provided an AIC of 4,338, and the best model using a combination of ILI and virus positive provided an AIC of 4,565. We also tried models with and without dummies for individual years; dummies for years were consistently better based on AIC.

Once it was determined that models with ILI and yearly dummies were best, we used a stepwise selection method to identify significant trend and seasonal terms in ILI models, separately for each age group and cause of death. Only terms with a p-value <0.05 were included in final models. Model fits were good, with correlation between predicted and observed values ranging between 0.56-0.93 (figure 2, figure S1).

Baseline mortality was predicted by setting influenza activity indicators to zero, following:

Baseline(t)= β_0_ + β_2*_t + β_3*_t^2^ + β_4_ t^3^ + β_5*_sin(2*Pi*t/52.17) + β_6*_cos(2*Pi*t/52.17) + β_7*_sin(3*Pi*t/52.17) + β_8*_cos(3*Pi*t/52.17) + β_9*_sin(4*Pi*t/52.17) + β_10*_cos(4*Pi*t/52.17) ) + β_11*_sin(6*Pi*t/52.17) + β_12*_cos(6*Pi*t/52.17) + β_13*_sin(8*Pi*t/52.17) + β_14*_cos(8*Pi*t/52.17) + β_15*_sin(10*Pi*t/52.17) + β_16*_cos(10*Pi*t/52.17) + β_17*_sin(12*Pi*t/52.17) + β_18*_cos(12*Pi*t/52.17)

Note that the baseline model only comprised significant trend and seasonal terms selected from the stepwise selection of age- and cause-specific models; we provide here the full equation for reference. The number of excess deaths due to influenza was defined as the difference between predicted number of deaths and baseline number of deaths. The seasonal number of excess deaths due to influenza was summed for each respiratory season. All respiratory seasons ran from July to June, except for the pandemic season which ran from May 2009 to May 2010, and hence included a full 12-month period during which influenza was not always active. Since the A/H1N1 pandemic virus circulated sporadically before September 2009 and after February 2009, we conducted a sensitivity analysis considering a shorter pandemic season running from September 2009 to February 2010. We obtained mortality estimates within 2% of estimates derived under the longer pandemic period.

Age-standardized seasonal excess mortality rates per 100,000 due to influenza were calculated, using the population of 2009 as reference, so that we could compare different seasons with different population age structure. Confidence intervals for seasonal excess mortality estimates were calculated based on the variance of regression parameter estimates.

We also calculated the proportion of death attributable to influenza, separately for each season, mortality, outcome and age group. For instance for respiratory deaths in the A/H1N1 pandemic season, we divided the number of influenza-associated excess respiratory deaths derived by the Poisson model for May 2009-May 2010, by the total number of respiratory deaths during May 2009-May 2010.

We conducted supplementary analysis considering 10 different age groups (0-4, 5-14, 15-24, 25-34, 35-44, 45-54, 55-64, 65-74, 75-84, ≥85 yrs). We also tried Poisson models with identity links rather than log link; estimates were within 3% of each other.

SAS software version 9.2 (SAS Institute, Inc., Cary, NC) proc Genmod was used for all analyses.

***Serfling method***

The Serfling approach is a traditional seasonal linear regression model to estimate excess mortality in the absence of information on influenza activity [14]. The model represents the expected level of mortality in the absence of mortality, hence a “baseline”.

We first defined influenza epidemic weeks by applying a Serfling model to weekly all-age mortality rates specifically attributed to influenza, excluding values for the period December to April [14,17] following:

E(Y*_t_*) = β_0_ + β_1*_t + β_2*_t^2^ + β_3_ t^3^ + β_4*_sin(2*Pi*t/52.17) + β_5*_cos(2*Pi*t/52.17) where Y_t_ represents weekly number of flu-specific deaths at week t, t. t^2^ and t^3^ are linear, quadratic and cubic terms for time trends, sin and cos are harmonic terms representing annual periodicity,

A week was considered as epidemic if the observed influenza mortality rate exceeded the upper 95% limit of the prediction interval ,calculated as predicted baseline + 1.96 standard deviation. Next, we applied a similar model to each age group and cause of death, after, setting values for epidemic weeks to missing.

The number of excess deaths attributable to influenza was estimated as the difference between the observed and baseline number of deaths during epidemic weeks. We summed weekly excess deaths for each respiratory season to provide seasonal estimates and age-standardized seasonal excess mortality rates per 100,000.

The Serfling approach produced estimates of 514 excess respiratory and 1,008 cardio-respiratory excess deaths for the 2009-10 pandemic (death rates of 0.82 and 1.61 per 100,000, table 1).

**Years of life lost (YLL) estimation**

We used the YLL approach to integrate the age distribution of excess deaths in mortality burden estimates, as previously used for influenza [[4](#_ENREF_4), [39](#_ENREF_39), [47](#_ENREF_47), [48](#_ENREF_48)]. Specifically, we multiplied the age-specific number of excess deaths by the age-specific life expectancy in 2009 in France [[49](#_ENREF_49)]. To provide national seasonal estimates of YLL, we summed age-specific estimates of YLL across all age groups (Table 2).
